# Application of Modular Architectures in the Medical Domain - a Scoping Review

**DOI:** 10.1007/s10916-025-02158-3

**Published:** 2025-02-18

**Authors:** Franziska Bathelt, Stephan Lorenz, Jens Weidner, Martin Sedlmayr, Ines Reinecke

**Affiliations:** 1 Thiem-Research GmbH, Thiemstraße 111, 03048 Cottbus, Brandenburg Germany; 2 Carl Gustav Carus Faculty of Medicine, Center for Medical Informatics, Institute for Medical Informatics and Biometry, Technische Universität Dresden, Fetscherstr. 74, 01307 Dresden, Saxony Germany

**Keywords:** Microservice, Service-oriented architecture, Healthcare, Health information interoperability, Scalability

## Abstract

The healthcare sector is notable for its reliance on discrete, self-contained information systems, which are often characterised by the presence of disparate data silos. The growing demands for documentation, quality assurance, and secondary use of medical data for research purposes has underscored the necessity for solutions that are more flexible, straightforward to maintain and interoperable. In this context, modular systems have the potential to act as a catalyst for change, offering the capacity to encapsulate and combine functionalities in an adaptable manner. The objective of this scoping review is to determine the extent to which modular systems are employed in the medical field. The review will provide a detailed overview of the effectiveness of service-oriented or microservice architectures, the challenges that should be addressed during implementation, and the lessons that can be learned from countries with productive use of such modular architectures. The review shows a rise in the use of microservices, indicating a shift towards encapsulated autonomous functions. The implementation should use HL7 FHIR as communication standard, deploy RESTful interfaces and standard protocols for technical data exchange, and apply HIPAA security rule for security purposes. User involvement is essential, as is integrating services into existing workflows. Modular architectures can facilitate flexibility and scalability. However, there are well-documented performance issues associated with microservice architectures, namely a high communication demand. One potential solution to this problem may be to integrate modular architectures into a cloud computing environment, which would require further investigation.

## Introduction

The advancing digitization creates a growing need for more flexible architectures and methods, to increase reusability, maintainability, replaceability and scalability [1]. In industry, this trend becomes obvious as cloud-native application have gained increasing popularity since 2015 [2]. Blueprints for those applications are service-oriented architectures (SOA) [3] and microservice architectures [1]. Both types of architectures, which are summarized below as *modular architectures*, are based on the encapsulation of functionality in smaller components called services, which are assembled to form a more complex functionality. The components themselves are interchangeable and reusable. Their compact size makes them easy to maintain and scalable. The main difference between the two architecture types lies in the definition of the services. In microservice architectures, the components are developed and deployed independently so that they operate autonomously. In SOA, services may depend on the functionality of other services and may not function independently. In both types of architectures, the complexity that exists in monolithic systems is transferred to the increasing complexity of service composition and service discovery [4].

However, as the Global Digital Health Index [5] illustrates, this trend has yet to be reflected in the healthcare sector. Instead, it is characterised by the utilisation of self-contained, monolithic information systems and data silos [6]. These monolithic systems frequently exhibit the lack of, or high cost of, interfaces, which is contrary to the principle of interoperability. The openness and flexibility of *modular architectures* can facilitate technical and organisational interoperability by providing access to interfaces and reducing costs through the cost-efficient, needs-based provision of digital support services. According to Lehne et al. [7] interoperability is a fundamental requirement for digital (intersectoral) medicine as well as international cooperation and research. The necessity for interoperability gives rise to the question of why monolithic systems continue to prevail as the norm in the medical sector.

Existing studies are of little help in identifying barriers to the productive use of *modular architectures*. Javandi and Kashanian [8] addressed the broader issue of the Internet of Things and its application in the medical domain. They mainly focus on sensors and sensor networks and pointed out that the standards used in SOA can be helpful in the context of the Internet of Things. Al-Jaroodi, Mohamed, and Abukhousa [9] identified the main goals for Health 4.0, following Industry 4.0, and presented their idea of service-oriented middleware to improve efficiency and effectiveness in identifying and using services. A more related review was provided by Avila et al. [10], which deals with applications of SOA in the field of home care. The authors summarized existing functionalities and solutions for security questions.

To the best of our knowledge, a comprehensive overview of the use and maturity level of *modular architectures* in the health care sector, as well as their existing barriers, is still absent. However, understanding the principles and guidelines for a successful transition from monolithic systems to modular ones is crucial for achieving optimal outcomes. In this paper, we investigate the extent to which the application of service-oriented and/or microservice architectures within the medical sector has been addressed in current literature. We identify opportunities, challenges, potential solutions, and the maturity level of these solutions. We address the following research questions, which form the knowledge base for the initial transformation steps :

1. Has there been an increase in the use of service-oriented or microservices architectures in the medical field over the past five years, suggesting an increasing popularity in the medical field?
2. What are best practices from countries where service-oriented or microservices architectures are routinely deployed?
3. What needs to be considered when implementing a *modular architecture* in the medical domain?

## Methods

For the review process, we followed the Preferred Reporting Items for Systematic Reviews and Meta-Analyses (PRISMA) statement and used the template for “PRISMA 2020 flow diagram for new systematic reviews which included searches of databases and registers only” to document our findings [11]. Potential databases for the literature search were discussed, and it was decided that PubMed and WebOfScience would be used. The decision to prioritize PubMed was influenced by its specialized focus in the medical field, while WebOfScience was selected due to its comprehensive indexing of major relevant journals (e.g., IEEE, Elsevier, etc.). We conducted literature searches on 4th of February 2022, 12th of August 2022, 9th of December 2022, and 1st of August 2023 in the databases PubMed and WebOfScience. We used the search strings listed in Table 1 and set the filter to the time period 2017-2022. Manual validation was performed to ensure the efficacy of the indexing process for IEEE Xplore in Web of Science, ruling out the possibility of additional publications being identified. The search string was designed with a specific focus on the Title and Abstract, and it was decided that the abbreviation “SOA” would not be used due to the aforementioned design and the potential for unrelated results. Secondly, it is imperative to note that journals do not accommodate abbreviations within the abstract section. Consequently, the use of abbreviations within the search string does not lead to additional publications. To address the issue of duplicate detection between search periods, an R script was developed to calculate the discrepancy between the previous and subsequent search results. This R script is available in the supplementary files.

**Table 1:** Search string definition

Data base	Search string
PubMed	(((Service-oriented Architecture[Title/Abstract]) OR
	(microservice[Title/Abstract]) OR (micro-service[Title/Abstract]))
	AND ((health*[Title/Abstract]) OR (medic*[Title/Abstract])))
WebOfScience	(TS=(service-oriented architecture) OR TS=(microservice)
	OR TS=(micro-service)) AND (TS=(health* ) OR TS=(medic*))

In an interdisciplinary team (computer scientists, business computer scientists, public health specialist) we defined inclusion (Table 2) and exclusion criteria (Table 3) for the title-abstract and full text. It should be noted, that the exclusion reasons are not unique. Thus one publication can have more than one exclusion reason.

For the title-abstract screening we uploaded the search results to rayyan [12]. We used the blind mode to independently include or exclude publications by two persons. After all publications have been tagged we switched off the blind mode and personally discussed conflicts. All resulting included publications underwent the full text screening. For this we started a new review on rayyan comprising only this subset of publications. We conducted a second blind review based on the full texts in accordance to the title-abstract screening process. Based on the remaining full texts, we extracted content using the extraction parameters listed in Table 4.

**Table 2:** Inclusion criteria

Type	Description
Technology	Publication addresses an architecture or infrastructure that follows
	the SOA paradigm or the paradigm of microservices
Application	*Modular architectures* are addressed in a medical context
	(including those where the validation was carried out on a
	medical use case)

**Table 3:** Exclusion criteria

Label	Description
no_medic	Publication does not deal with the medical field
medic_mentioned	Publication mentions medical field only as potential
	application area
no_soa	Publication does not deal with a modular
	infrastructure (e.g. service only as an “offer”)
no_architecture	Publication only deals with one component (Software as
	a Service) without addressing the combinations
soa_mentioned	Publication only mentions service architecture as a
	potential area of application
foreign_language	Publication is available in a language other than German
	or English
wrong_publicationtype	Publication is just an abstract, keynote or tutorial

**Table 4:** Extracted parameters

Parameter	Description
Author	List of authors
Publication year	Year where the article has been published
Title	Title of the publication
Country	Country of the affiliation of the first author
Architecture type	Microservice OR service-oriented architecture
Application area	Intersectoral, home care, hospital or local practitioner
Level	Concept, proof of concept or routinely usage
Addressed problem	List of primary technical problems addressed in the article
Solution	List of solutions for the addressed problems
Problem list	List of open challenges/ limitations (e.g. security, privacy,$\ldots$)
Benefits	List of potential benefits (e.g. flexibility, reusability)

Based on the extracted data we performed a statistical analysis about the annual distributions e.g. of publications, architecture types, and application areas. We linked countries with maturity levels and we summarized problems, problem list and solutions. Statistical analysis and visualizations were performed via an R script. This can be found in the supplementary materials.

## Results

### Literature Search

**Fig. 1:**
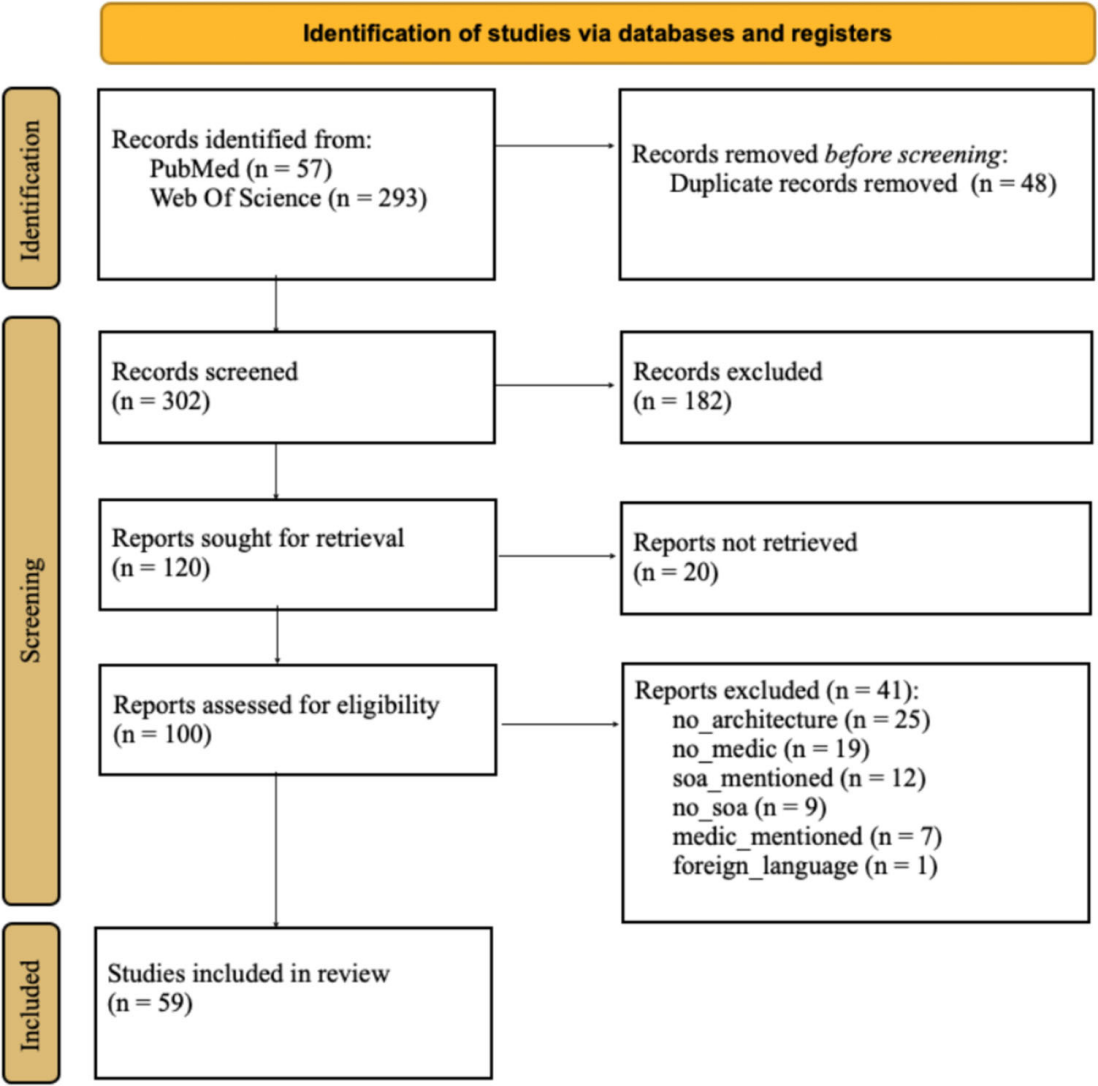
PRISMA: Identification process of relevant literature in accordance with [11]

The literature search resulted in 350 potentially relevant publications (Fig. 1). 48 of those were duplicates and were removed. In the process of the Title-Abstract-Screening additional 182 irrelevant publications and 20 further, potentially relevant but without access to full texts publications were removed. The remaining 100 publications underwent the full text screening process in which 41 were excluded, mainly as they do not address architectures nor the medical field. Finally, 59 publications were included and analyzed.

### Statistical Analyses

To answer research question 1 (RQ 1), statistical analyses were conducted.

**Fig. 2:**
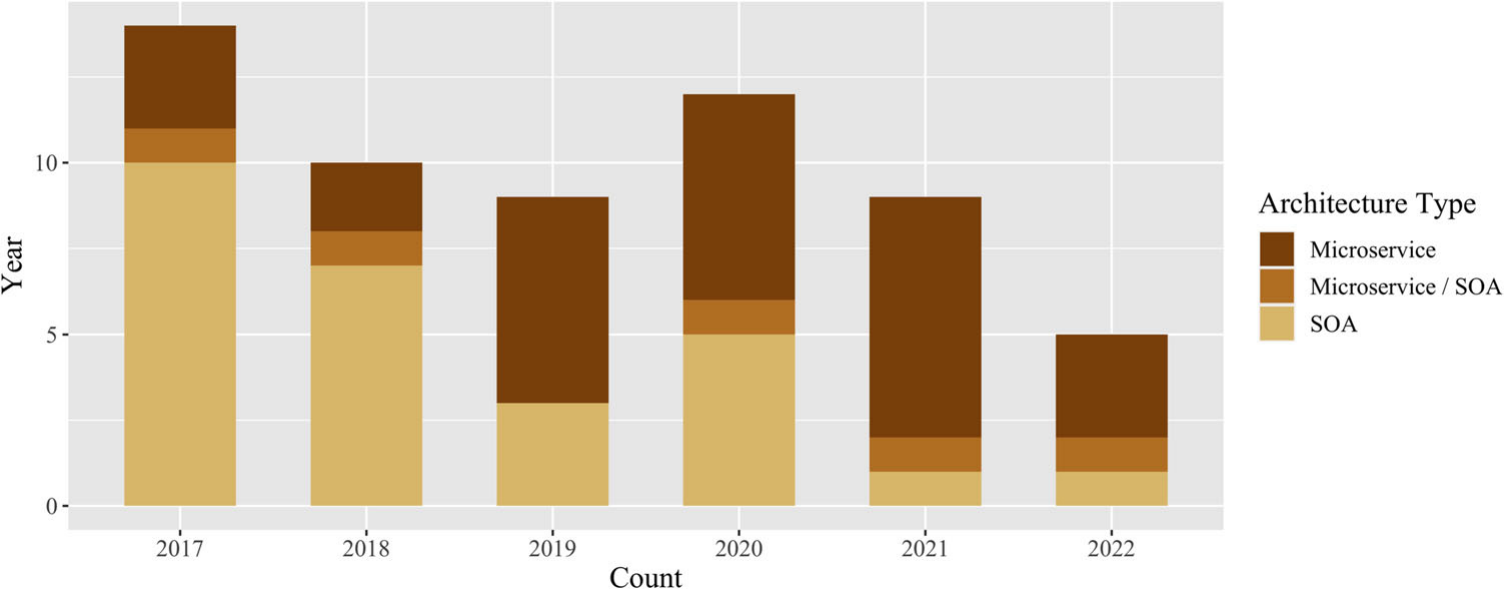
Annual distribution of publications on the different types of architectures

The analysis of the annual distribution of the 59 included publications with respect to the architecture type (Fig. 2) shows a decrease in the overall numbers, except for the year 2020. However the number of publications dealing with microservices increases compared to service-oriented architectures.

**Fig. 3:**
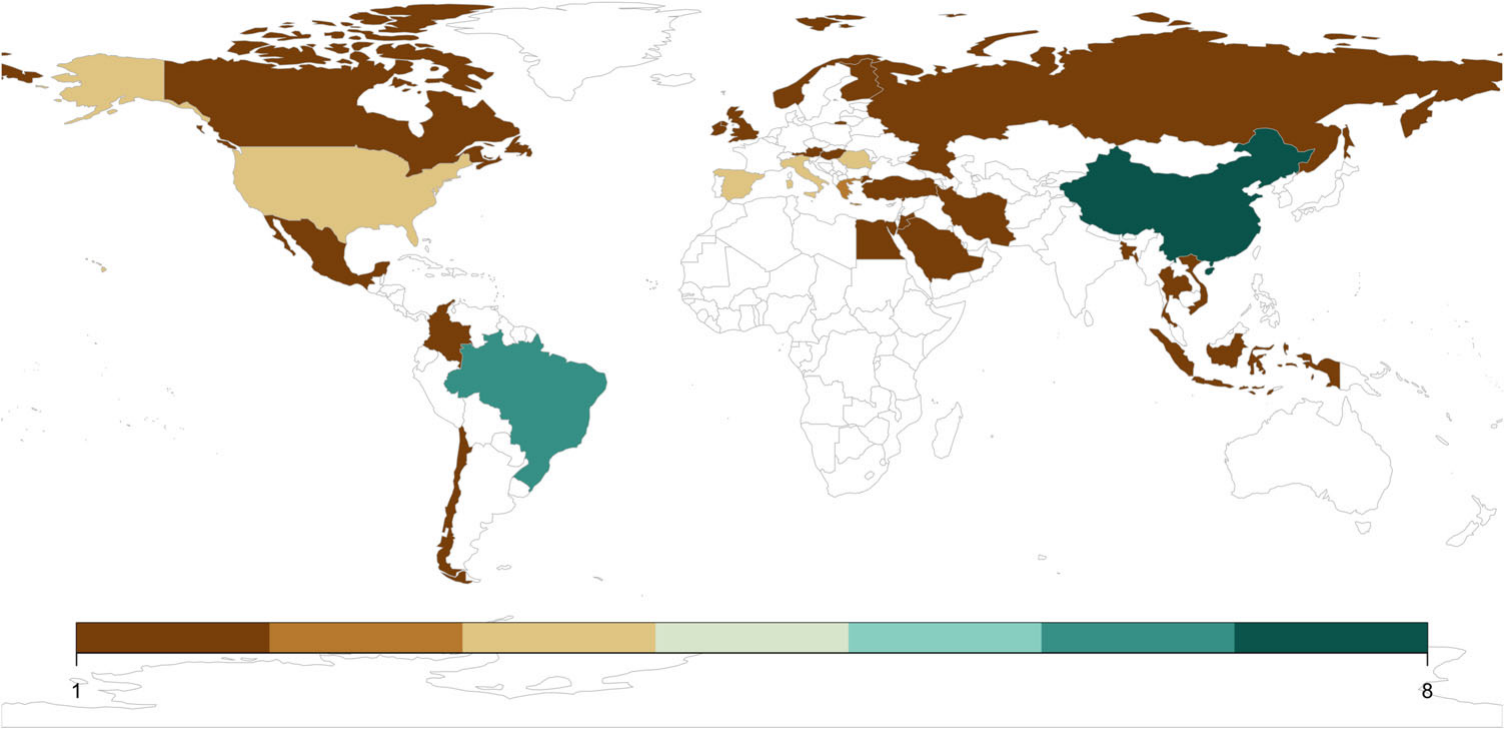
Overview over publishing countries. Colours represent the amount of publications that are associated with the corresponding country

The analysis of countries (Fig. 3), that can be associated with the publications, shows a dominance from China and Brazil, followed by USA, Spain, Italy and Romania. Countries like Estonia, Denmark, Switzerland, France and Germany are not among the publishing countries.

**Fig. 4:**
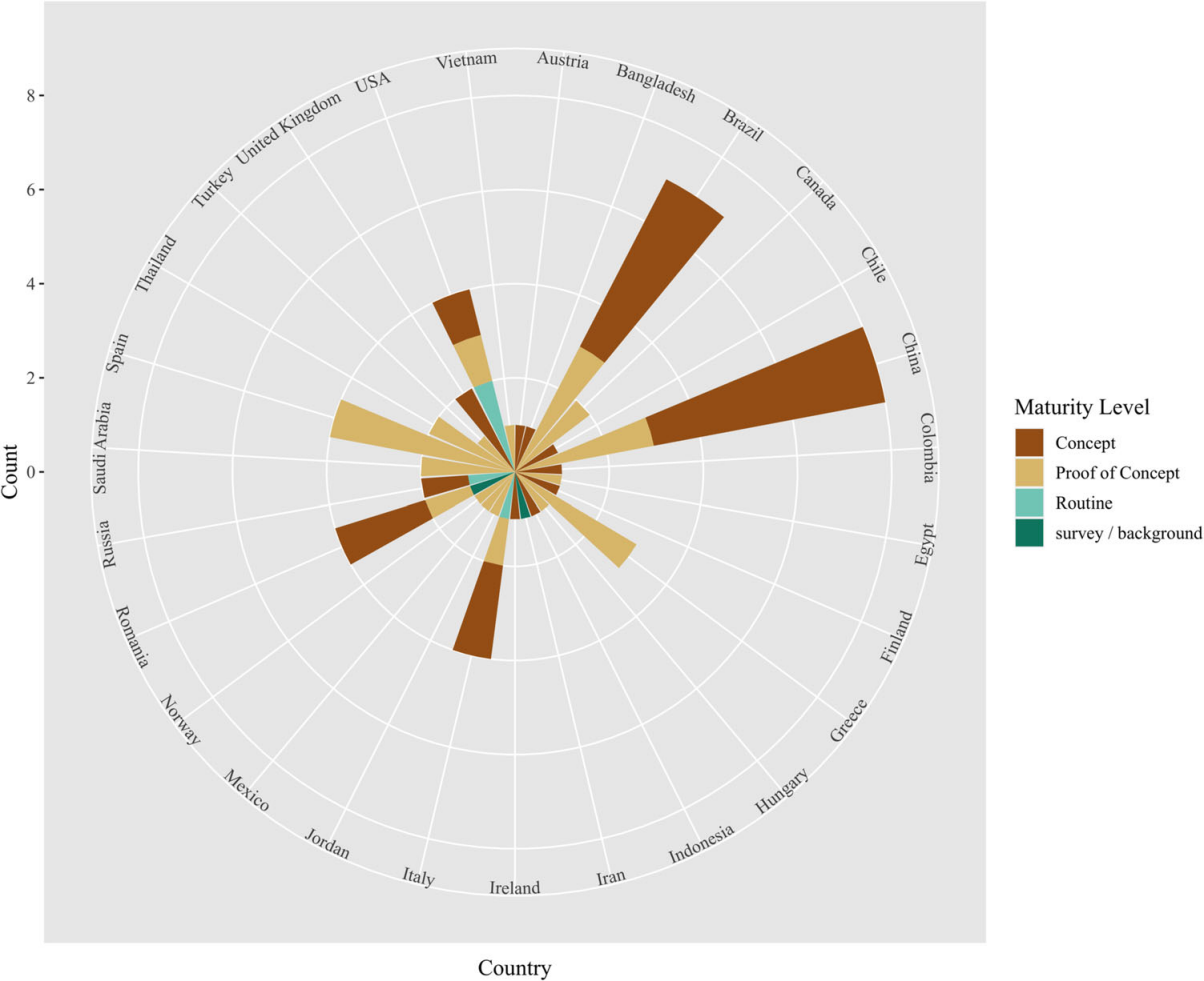
Overview over the maturity level of solutions and their association with publishing countries

Focusing on the maturity level of the proposed solutions (Fig. 4), it is obvious that most of the publications deal with concepts or proof of concepts. The latter may be available in routine care in the near future. *Modular architectures* are already in routine use in the United States [13, 14], Russia [15], and Italy [16]. The publications range from standardized data sharing platforms [15, 16] via the realization of legal requirements regarding security on each level [13] to support decision support and separating technical from usage level [14].

### Considered Aspects for Implementation

To answer the research questions RQ 2 and RQ 3, extracted data has been analysed. The recommendations that are extracted from the findings stated below focussing on the countries where service-oriented or microservice architectures are routinely deployed (RQ2) are part of the discussion.

The extraction shows a concentration on five main aspects that are addressed in the publications and described below. Some publications address different topics and are therefore referenced multiple times below. The extraction table can be found in the supplementary files.

#### Interoperability and Data Exchange

One of the most addressed problems is interoperability and data exchange. Publications dealing with this topic present the use of Health Level Seven (HL7) standards [13, 16–20] and HL7 Fast Healthcare Interoperability Resources (FHIR) [15, 21–23] as possible solutions to achieve syntactic and semantic interoperability.

To support data exchange and technical interoperability authors propose to use SOA implementation standards, such as WSDL [24] and RESTful interfaces [20, 25, 26] .

Organizational interoperability can be achieved by semantic workflows based on an ontology database [27] or by applying ISO norms, such as ISO/IEC IS 10746 - Open Distributed Processing [28] within the *modular architecture*.

#### Security and Privacy

The second most frequently addressed subject was security and privacy on different levels. At a superordinate level some publications refer to the use of the HIPAA (Health Insurance Portability and Accountability Act) security rule [13, 17] or to the provision of separated infrastructures (e.g. virtual machines and services) to prevent an unauthorized access [29, 30]. On a more specific level, authentication/authorization [22, 31–34] and message de- and encryption [35], such as Advanced Encryption Standard (AES) [19] or Message Authentication Codes (MAC) [36], are listed as solutions for security and privacy issues. Audit trails (e.g. IHE ATNA Profile) [18] or audit logs [37] further enhance the transparency for security breaches.

#### Scalability, Flexibility, and Reusability

To support scalability, flexibility and reusability, monolithic systems should be divided into small components, i.e. services, that can act individually [38]. Various architectures for different medical use cases are described in the literature such as Digital Biobanks [29] and blood centers [39], Rehabilitation [40], prenatal care [25], chronic disease [22, 41], Diabetes mellitus [30, 42], Disaster Management [43], Childhood obesity [44], Community Health Care [45], student-run clinics [46], Family Doctor Systems [47], and Epidemic prevention [48].

They differ mainly in terms of the services involved, but the basic idea remains the same. Smaller service implementation allow for an exchange and thereby reduce the probability of failure for the overall system. They are easily maintainable and can be reused in different settings [49]. The modular structure allows an easy expansion of the functionality via new combinations of services, and it accommodates a variety of users and user contexts through multiple executions of each service [50, 51].

#### Design and Service Discovery

For a flexible exchange of services, design of the architecture and automated service discovery is crucial. The design process requires multiple perspectives on the architecture [52], such as stakeholder participation [53, 54]. Alternatively, a general framework can serve as a reference model [55] or a foundation for development [14, 56–59].

For automated service discovery, the contextual use of the service to be identified is important. This comprises the knowledge about user and use cases [60, 61], the process involved [62] as well as interfaces [23] and Quality of Service properties, such as low latency [63, 64] that are required.

#### Performance

The *modular architecture* and the approaches for automated service discovery allow for the use case specific identification of suitable services. Thus, SOA or microservices can be used to solve real-world performance issues. Examples for this are real-time data processing for monitoring in the context of ambient assisted living [65], of a smart medicine box [66] and monitoring by activity trackers [67].

However, SOA and microservices need higher orchestration effort that must be taken into account when designing the architecture. Performance evaluations regarding SOA and microservice architectures indicate a higher communication efficiency for microservices [68]. However, the performance seems to be dependent on the amount of communicating services and the individual workload of each service. To overcome this, an automated replication in accordance to the workload and an individualization of the communication to other services, to prevent bottlenecks should be supported [69].

## Discussion

### Implication of Findings

The review shows an increased use of microservices compared to SOA, which implies a paradigm shift to an encapsulation of autonomic functionalities. Although microservices have some drawbacks, like a higher need for security and communication overhead [70], their flexibility and scalability seem to be more advantageous. The scalability aspect requires the availability of sufficient services that can be composed. To enable the discovery of sufficient services and their composition, the use of standards is necessary. For communication, HL7 and HL7 FHIR seem to be the leading standards. Both are already used in productive applications in the United States [13], Russia [15], and Italy [16]. RESTful interfaces and standard protocols as HTTP support technical data exchange. For security and privacy aspects, message en- / decryption, authentication and authorization standards, as well as the HIPAA security rule [71] should be considered [13].

It seems that the pandemic, which started in 2020, was a driver for an increased research effort to support digitalization and communication in the medical sector. However, in last five years only concepts and proof of concepts were published. The way into routine care is still an open issue.

### Recommendations

From the four publications that present routinely used architectures we can learn the following major aspects (RQ 2):

First, the legal requirements must be taken into account and comprehensively fulfilled. Since these requirements differ from country to country, it is advisable to focus on national framework conditions. Here, an analysis of the applicability of the HIPAA security rule [71] should be considered. In addition, a comprehensive analysis of commercial products in terms of their suitability for use should be performed. A deep dive into documentation is required here, even if compliance with national requirements is indicated by the manufacturer. Combining compliant products could compromise overall system compliance [13].

Second, routinely used platforms for data exchange, data harmonization and data analysis require standardized data, which implies the availability of implementation guides for all data sets to be integrated [15, 16]. It is important that these implementation guides are standardized (nationally) and that all stakeholders (e.g., data providers, data users) are required to use this specification. Gazzarata et al. [16] suggest to actively participate in (HL7 FHIR) working groups responsible for standardization.

Third, besides the technical aspects acceptance is crucial. For medical staff, that represent the major user group, it is uninteresting how data is stored, calculations are done and how the technical architecture is defined. Platforms must provide an easy to use user interface which is highly adaptable to the user’s need. Additionally, it must be integrated into the work processes of the medical staff and the access must be granted through all relevant devices, to increase the research efficiency [14].

Fourth, increased operation complexity and performance slowdown are known drawbacks of microservice architectures [15, 70]. Concepts are needed to deal with federated logging analysis to support maintenance, accelerate distributed processing and transaction handling and to decrease communication overhead [15].

### Open Issues

It was our intention to identify potential solutions to this performance issue within the existing literature. However, the included publications addressed the issue of performance solely in the context of work performance among medical staff and in the context of application development. Therefore, the issue of performance in the context of *modular architectures* remains unresolved and requires further investigation. In light of the aforementioned statements, it can be assumed that this may be achieved by the construction and incorporation of *modular architectures* into a cloud environment, with the objective of facilitating the parallelisation of services for the purpose of enhancing performance, reducing maintenance and operational complexity, and enabling the automatic scaling of services. In this context, the development of performance test beds is proposed as a means of achieving comparability between architectures and approaches. Furthermore, the creation of safety test beds is recommended as a strategy to enhance confidence in the architectures employed in domains where safety is of paramount importance, such as healthcare.

## Conclusion

The implementation of service-oriented or microservice architectures can facilitate flexibility and scalability, thereby reducing the requisite development effort and time. In the context of healthcare, this facilitates the provision of an enhanced quality of care in rural areas. In order to achieve this, it is necessary to establish the requisite technical prerequisites in the form of a cloud infrastructure, within which the applications can be utilised by the relevant medical personnel. The most significant consideration is the necessity of standards for achieving syntactic and semantic interoperability. It is imperative to engage proactively with the relevant technical committees and working groups in order to ensure the availability and adequacy of standards. It is of the utmost importance to comply with the relevant legislative requirements, such as those pertaining to data security and data protection, for the regular use of such systems. It is essential to maintain a clear distinction between the technical and usage-related aspects in order to facilitate seamless integration into existing medical workflows and ensure widespread acceptance. The rapid evolution of the healthcare sector underscores the necessity for ongoing adaptation and refinement of *modular architectures*, thereby underscoring the need for future studies and advancements in this domain.

## Supplementary Information

The supplemental material [72] includes the following files:

1. Detect_Divs_Review-helper-final.zip: Helps to find the conjunction between two or more literature searches. Currently, PubMed and Web of Science is supported.
2. Results_Rayyan.zip: Includes all (sub) decisions of the review process
3. Kappa-Value-Preparation-final.zip: Is an R-script to determine the Kappa-Value of decision of two reviewers.
4. Extraction-final.csv: Is the final spreadsheet comprising all publications and the extracted aspects as final result of the review process.
5. SOAinMED_analysis.R: Is an R-script for the visualization of the extracted data

## Data Availability

See Supplemental Material.

## Code Availability

See Supplemental Material.
